# Carotid Plaques and Hypertension as Risk Factors for Cardiovascular Disease and All-Cause Mortality in Middle-Aged Adults

**DOI:** 10.3390/jcm13102804

**Published:** 2024-05-09

**Authors:** Vilma Dženkevičiūtė, Tadas Adomavičius, Gabrielė Tarutytė, Egidija Rinkūnienė, Vytautas Kasiulevičius, Jolita Badarienė

**Affiliations:** 1 Clinic of Internal and Family Medicine, Faculty of Medicine, Institute of Clinical Medicine, Vilnius University, LT-03101 Vilnius, Lithuania; tadas.adomavicius@mf.vu.lt (T.A.); vytautas.kasiulevicius@mf.vu.lt (V.K.); 2 Department of Research and Innovation, Faculty of Medicine, Vilnius University, LT-03101 Vilnius, Lithuania; gabriele.tarutyte@santa.lt; 3 Clinic of Cardiac and Vascular Diseases, Faculty of Medicine, Institute of Clinical Medicine, Vilnius University, LT-03101 Vilnius, Lithuania; egidija.rinkuniene@santa.lt (E.R.); jolita.badariene@santa.lt (J.B.)

**Keywords:** all-cause mortality, cardiovascular disease, carotid plaque, hypertension

## Abstract

**Background/Objectives**: Both hypertension and carotid atherosclerosis are independent risk factors for cardiovascular disease. We aim to investigate the synergistic effects of hypertension and carotid plaques on cardiovascular events and all-cause mortality. **Methods**: A follow-up study was conducted at the Preventive Cardiology Department of Vilnius University Hospital Santaros Klinikos between 2012 and 2021. The study recruited participants aged 40–65 who did not have overt cardiovascular disease (CVD) and were part of the Lithuanian High Cardiovascular Risk primary preventive program. The study collected demographic and clinical data, including an ultrasound assessment of carotid plaque. **Results**: The participants were monitored for 4–10 years for CVD events and all-cause mortality. Among 6138 participants, 954 (16%) experienced CVD events. The presence of carotid plaque on both sides was significantly associated with CVD events, myocardial infarction, and all-cause mortality. However, the combination of hypertension and carotid plaque did not significantly increase the risk for CVD events or all-cause mortality. **Conclusions**: The risk of CVD events or all-cause mortality was not significantly increased by the combination of hypertension and carotid plaque. Cardiovascular events depend on the extent of atherosclerosis in the carotid arteries.

## 1. Introduction

Cardiovascular diseases (CVDs) are a common cause of death worldwide, with increasing poor survival rates. Despite the decrease in age-specific cardiovascular death rates, absolute CVD mortality and morbidity keep increasing [1] because of population aging and overall population growth [2]. The number of CVD-related deaths rose from 12.1 million (95% UI: 11.4 to 12.6 million) in 1990 to 18.6 million (95% UI: 17.1 to 19.7 million) in 2019 [3]. Atherosclerosis, characterized by the accumulation of lipid-rich plaques within arterial walls, is the leading cause of vascular disease globally [4,5]. As atherosclerosis is a generalized disease affecting many arterial beds simultaneously, assessing carotid arteries creates an opportunity to mirror and track atherosclerotic disease [6,7,8,9]. Carotid artery plaque is a known predictor of coronary heart disease (CHD). Extensive cohort studies have consistently shown that individuals with atherosclerotic plaques face a 1.3- to 2.8-times-higher risk of CVD compared to those without plaques. The progression of total plaque area (TPA) is a strong predictor of stroke, death, and myocardial infarction (MI). Therefore, measuring TPA can be a better approach to treating arteries rather than focusing solely on risk factors, as it significantly reduces the risk of cardiovascular events [10,11,12,13]. High blood pressure (BP) was the leading risk factor for the overall global burden of disease in 2010 [14] and is a significant risk factor for the development of CVD [14,15,16]. Systolic and diastolic blood pressure show heterogeneous associations across various acute and chronic cardiovascular diseases and at different ages [17]. Longitudinal studies, genetic epidemiological studies, and randomized controlled trials have shown that raised BP is a significant cause of both atherosclerotic cardiovascular disease (ASCVD) and non-atherosclerotic CVD (particularly heart failure), accounting for 9.4 million deaths and 7% of global disability-adjusted life-years [14]. Hypertension can cause hypertension-mediated organ damage (HMOD) in different organs such as the brain, kidneys, heart, retina, and arteries. Detecting HMOD can help identify patients at high cardiovascular risk, especially middle-aged asymptomatic patients [16].

In theory, patients with both hypertension and atherosclerotic plaques may face an even higher risk of CVD events compared to those with only one of these risk factors [13,17]. However, scientific reports and empirical data that specifically examine the combined effects of hypertension, atherosclerotic plaques, and CVD, as well as their impact on all-cause mortality, are lacking. To address this knowledge gap, our study investigated whether both plaques and hypertension are associated with an increased incidence of CVD events and all-cause mortality. Additionally, we aimed to investigate the relationship between the spread of atherosclerosis and the occurrence of cardiovascular disease events, as well as total mortality.

## 2. Methods

### 2.1. Study Design and Population

The dataset of this study consisted of patients who participated in the Lithuanian High Cardiovascular Risk (LitHiR) primary prevention program. This state-funded program started in 2006 in Lithuania, focusing on preventing early atherosclerosis development in middle-aged individuals with high cardiovascular risk. The program included individuals between 40 and 65 years of age diagnosed with metabolic syndrome and investigated between 2006 and 2023 in Vilnius University Hospital Santaros Klinikos. Metabolic syndrome was defined as meeting three or more of the following criteria: waist circumference ≥ 102 cm in men and ≥88 cm in women; systolic blood pressure (SBP) ≥ 130 mmHg and/or diastolic blood pressure (DBP) ≥ 85 mmHg; fasting plasma glucose ≥ 5.6 mmol/L or type 2 diabetes mellitus; triglyceride (TG) concentration ≥ 1.7 mmol/L; and high-density lipoprotein (HDL) cholesterol < 1.03 mmol/L in men and <1.29 mmol/L in women. The patients did not have overt cardiovascular disease. The data for this study were collected prospectively and standardized within the principal LitHiR program. Essential variables for the study were extracted from this data, including smoking, LDL, HDL, total cholesterol (TC), SBP, DBP, fasting glucose, heart rate BMI, and carotid ultrasonography. Only subjects with recorded values for all of these variables were included in the study to ensure the comprehensiveness and reliability of the risk stratification. Patients with previously diagnosed coronary artery disease, silent myocardial ischemia, a transient ischemic attack, an ischemic and hemorrhagic stroke, peripheral artery disease, active oncological disorders, advanced kidney or hepatic failure, chronic or persistent arrhythmias, severe psychiatric disorders, drug addiction, or pregnancy were excluded from the study.

### 2.2. Data Collection

Demographic data, encompassing age and sex, were collected. Smoking status was recorded based on participants’ self-reports, categorized into four groups: (a) non-smoker; (b) smokes less than 10 cigarettes/day; (c) smokes 10 or more cigarettes/day; and (d) quit smoking. Blood pressure was measured using a manual sphygmomanometer (Riester precisa^®^, Jungingen, Germany). A cuff was positioned on the upper arm while sitting, according to all recommendations of the European Society of Cardiology Guidelines for managing arterial hypertension [18]. A range of different clinical parameters essential for cardiovascular health were assessed. These parameters included heart rate, body mass index (BMI), TC, HDL cholesterol level, LDL cholesterol level, TG concentration, intima-media thickness (IMT) of arterial walls, presence of atherosclerotic plaques, and a history of stroke and myocardial infarction among the subjects.

### 2.3. Carotid-Cranial Plaque Assessment

All study participants underwent carotid duplex ultrasound. Carotid ultrasonography was performed on both sides of the neck with the participant in the supine position using the Art.Lab system (Esaote Europe B.V., located in Maastricht, The Netherlands). The intima-media thickness within the common carotid artery was measured automatically six times over a segment of at least 15 mm. A plaque was defined according to the latest Mannheim Carotid Intima-Media Thickness and Plaque Consensus version. Carotid artery plaque is defined as a localized structure that encroaches into the arterial lumen by at least 0.5 mm or 50% of the surrounding intima-media thickness (IMT) value or demonstrates a thickness exceeding 1.5 mm. The carotid artery was divided into four segments bilaterally: common carotid artery, carotid bifurcation, internal carotid artery, and external carotid artery. We used cross-sectional sweeps to evaluate the presence of plaque in each segment. Finally, points for all segments on both sides were summarized to achieve a total carotid plaque score. The carotid plaque score was calculated during statistical analysis, and the operators were blinded to the participant’s cardiovascular morbidity/history.

### 2.4. Follow-Up and Outcomes

Participants were followed up throughout 2021 for the primary outcome of incident ischemic stroke (International Classification of Diseases, Tenth Revision [ICD-10], code I63.x)—myocardial infarction—and two secondary outcomes: composite endpoint MACE (nonfatal ischemic stroke [code I63.x], nonfatal myocardial infarction [code I21.x], and cardiovascular death [all codes I and R96]) and all-cause mortality. The outcome variables were based on individual-level data from the mandatory Lithuanian Patient Registry (LPR) and the Lithuanian Cause of Death Registry.

### 2.5. Statistical Analysis

All analyses were performed using R statistical software (version 4.2.2). Descriptive statistics were computed for demographic information. Data were expressed as mean ± standard deviation for continuous variables and as absolute and percentage values for categorical variables. Continuous variables were analyzed using a Student’s *t*-test or Mann–Whitney U test, and categorical variables with Pearson’s chi-square test. The normality of data was a test using the Shapiro–Wilk test. Normally distributed data were analyzed using one-way ANOVA, and non-normally distributed data were analyzed using Kruskal–Wallis tests. Cox proportional hazards regression was employed to estimate the risks of events, calculating hazard ratios (HR) and 95% confidence intervals (CI). A *p*-value < 0.05 was considered significant.

## 3. Results

### Baseline Characteristics of the Study Population, CV Events, and All-Cause Mortality

The data of 6138 participants were analyzed, of which 3571 were males (57%) and 2567 were females (43%). Among the total subjects, 5184 (84%) had no CV events, while 954 (16%) experienced CV events (49 participants (5.14%) had a MI, 128 participants (13.42%) had a stroke and 18 individuals (1.89%) experienced CV death). A comparison was conducted between the two cohorts (with and without CV events). The median age of patients with CV events was slightly higher than those without; this difference was statistically significant. There was a higher percentage of males in the group with CV events than those without, which was statistically significant. The distribution of smoking habits between the two groups was not significantly different. A higher percentage of patients with CV events had diabetes compared to those without, indicating a significant association. TC, LDL-C, and HDL-C levels showed small but statistically significant differences between the two groups. Patients with CV events had a higher median carotid intima-media thickness. The two groups’ statistically significant differences were not found in systolic or diastolic blood pressure. The group without cardiovascular (CV) events had a higher prevalence of carotid plaque on one side. In comparison, the group with CV events had a significantly higher prevalence of carotid plaque on both sides. The results are presented in Table 1.

**Figure 1:**
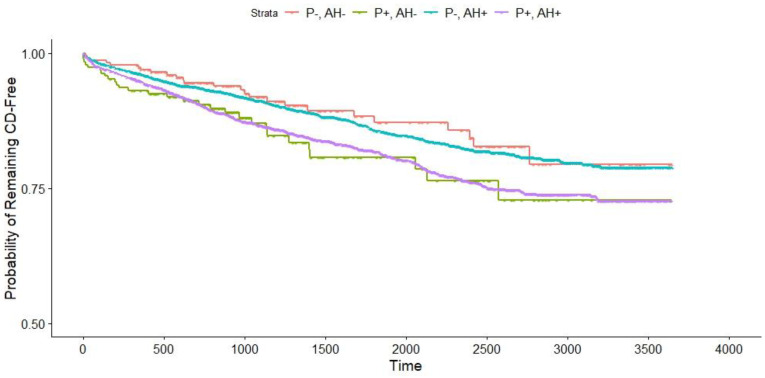
Cardiovascular events. Abbreviations: P-, AH- no hypertension or carotid plaque; P+, AH- carotid plaque only; P-, AH+ hypertension only; P+, AH+ hypertension with carotid plaque; CD cardiovascular diseases.

Participants were also categorized into four groups: (a) no hypertension and no plagues (b) only hypertension without plagues (c) only carotid plaque and no hypertension; and (d) hypertension with carotid plaque. These groups were compared to patients who did not have hypertension or carotid plaque. The results showed (Figure 1) that participants with carotid plaque alone and those with hypertension and carotid plaque exhibited the same risk of cardiovascular events. On the other hand, participants without carotid plaque but with hypertension had the same all-cause mortality rate as those without both hypertension and carotid plaque. However, the small number of events in the study meant that the results did not have statistical significance. The detailed results of the multivariable Cox proportional hazards regression analysis can be found in Table 2. Only carotid plagues on both sides are statistically significant for cardiovascular events, myocardial infarction, and all-cause mortality.

## 4. Discussion

Both hypertension and carotid atherosclerosis are risk factors for cardiovascular disease. However, it is essential to understand that they are part of a broader network of metabolic issues. This cluster includes factors such as dyslipidemia, diabetes, obesity, chronic inflammation, arteriosclerosis, and unhealthy lifestyle habits [19,20,21].

According to our data, there were more cardiovascular events among males than females. This may be due to biological, lifestyle, and sociocultural differences. Men and women have different hormonal profiles that can affect cardiovascular health. Pre-menopausal women are generally protected against heart disease because of estrogen. Lifestyle factors such as smoking, alcohol consumption, and diet can increase the risk of cardiovascular disease. In our data, a higher percentage of males were smokers compared to females, which could contribute to the higher incidence of cardiovascular events in males. Sociocultural factors such as stress, occupation, and societal roles may also play a role. To reduce the risk of cardiovascular disease, it is important to consider these factors in developing interventions. Our study did not find a significant increase in CVD events, stroke, myocardial infarction, or all-cause mortality when hypertension and atherosclerotic plaques were combined. In contrast, a study by Wen Li [22] showed that the combination of hypertension and atherosclerotic plaques increased the risk of CVD events and all-cause mortality, particularly cerebral infarction, compared to those without these factors. This discrepancy may be explained by the fact that the Wen Li study was conducted with Asian subjects, who have a higher incidence of strokes. In contrast, our study includes Caucasian subjects who have a higher incidence of myocardial infarction.

Our findings challenge the common belief that the coexistence of these two conditions would synergistically increase the risk of adverse cardiovascular outcomes. It suggests that the interaction between hypertension and atherosclerotic plaques may be more complex.

Several large population-based studies confirmed the predictive role of carotid plaques in the development of CVD [23,24,25]. Interestingly, our study found that only the presence of carotid plaques on both sides significantly increases the risk for cardiovascular events, myocardial infarction, and all-cause mortality, except stroke. This finding underscores the importance of carotid plaques as a risk factor for adverse cardiovascular outcomes. It shows that the usefulness of carotid ultrasonography for the risk stratification of cerebral and cardiovascular disease is based on various aspects. Our study suggests that bilateral carotid plaques may be a stronger predictor of these outcomes than the combination of hypertension and atherosclerotic plaques. Our findings are consistent with previous research that has emphasized the significance of carotid plaques, particularly bilateral ones, in predicting cardiovascular events, myocardial infarction, and all-cause mortality. A study by Håkon Ihle-Hansen et al. [26] found that the presence of carotid plaque scores was a strong predictor of ischemic stroke and major adverse cardiovascular events. Another study by Li Hongwei also supports our findings. The study states that plaque properties, including location, number, density, and size, become more important risk predictors for cardiovascular disease [27].

These studies suggest that the presence of carotid plaques, especially on both sides, could be a stronger predictor of adverse cardiovascular outcomes than the combination of hypertension and atherosclerotic plaques. This allows us to conclude that high blood pressure is a factor in the occurrence and progression of atherosclerosis, and the manifestation of atherosclerosis complications is related to time and the spread of the process. This underscores the importance of comprehensive risk assessment that considers not only traditional risk factors such as hypertension but also the presence and extent of carotid plaques. Future observational or clinical studies are needed to clarify the role of bilateral carotid plaques as a predictor for adverse cardiovascular outcomes.

## 5. Conclusions

One of the key innovative ideas in this study was the focus on the combined impact of hypertension and carotid plaques, which could have enhanced risk assessment and management strategies if a significant relationship had been found. The combination of these risk factors did not result in a significant increase in risk beyond what would be expected. The occurrence of cardiovascular events depends not only on the presence of hypertension and plaques but also on the severity and distribution of atherosclerotic lesions. Therefore, future studies should analyze the specific characteristics of carotid plaques, such as thickness, composition, degree of stenosis, and location, to refine risk assessment. Although clinicians should continue to address hypertension and monitor carotid health, the mere coexistence of these factors may not necessitate drastic management changes. Individualized risk assessment remains crucial, considering other variables such as lipid profiles, lifestyle factors, and comorbidities. However, this study is not without limitations. The sample was limited to participants aged 40–65 who did not have overt cardiovascular disease and were part of the Lithuanian High Cardiovascular Risk primary preventive program. Therefore, the findings may not be generalizable to other populations. Additionally, the study relied on ultrasound assessment of carotid plaque, which may not capture the full extent of atherosclerosis.

Future research should explore other potential synergistic effects between different risk factors for cardiovascular disease. It would also be beneficial to replicate this study in different populations and with more comprehensive measures of atherosclerosis. This could provide a more complete understanding of the risk factors for cardiovascular disease and inform more effective prevention and treatment strategies.

## Figures and Tables

**Table 1:** Baseline characteristics of the study population and incidence rate of adverse outcomes.

Characteristic	Population for Analysis, n = 6138		
	Without CV Event, n = 5184	With CV Event, n = 954	*p*-Value
**Follow-up** (years)	6.03 (4.27, 8.94)	8.38 (5.65, 9.55)	<0.001
**Age** (years)	53 (48, 58)	54 (50, 59.75)	<0.001
**Sex:**			
Male (%)	2964 (57.18)	607 (63.63)	<0.001
Female (%)	2220 (42.82)	347 (36.37)	
**Smoking:**			
Non-smoker (%)	3625 (70.62)	651 (68.60)	0.5
<10 cig./day (%)	538 (10.48)	106 (11.17)	
≥10 cig./day (%)	643 (12.53)	122 (12.86)	
Quit smoking (%)	327 (6.37)	70 (7.38)	
Unknown	51	5	
**Heart rate** (bpm)	66 (60, 73)	65 (59, 73)	0.4
**FBG** (mmol/l)	6.02 (5.66, 6.55)	6.01 (5.6, 6.63)	0.6
**Systolic BP** (mm Hg)	138 (129, 148)	138 (129, 149)	0.8
**Diastolic BP** (mm Hg)	84 (77, 91)	83 (76.25, 90)	0.3
**PAH:**			
Yes (%)	4818 (92.94)	900 (94.34)	0.12
No (%)	366 (7.06)	54 (5.66)	
**BMI**	31.55 (28.93, 34.6)	31.59 (28.72, 34.67)	0.8
**Diabetes**:			
Yes (%)	888 (17.13)	213 (22.33)	<0.001
No (%)	4296 (82.87)	741 (77.67)	
**TC** (mmol/l)	6.2 (5.37, 7.12)	6.32 (5.49, 7.3)	0.014
**LDL-C** (mmol/l)	4.03 (3.26, 4.79)	4.1 (3.33, 4.91)	0.017
**HDL-C** (mmol/l)	1.16 (0.99, 1.37)	1.13 (0.97, 1.34)	0.02
**CIMT**	659.5 (596.5, 731)	671.75 (602.62, 742.88)	0.004
**Carotid plaque:**			
Yes (%)	2438 (47.03)	431 (45.18)	<0.001
No (%)	2746 (52.97)	523 (54.82)	
**Carotid plaques on both sides:**			
Yes (%)	1008 (19.44)	222 (23.27)	0.007
No (%)	4176 (80.56)	732 (76.73)	
**MI:**			
Yes (%)	0 (0)	49 (5.14)	-
No (%)	5184 (100)	905 (94.86)	
**Stroke:**			
Yes (%)	0 (0)	128 (13.42)	-
No (%)	5184 (100)	826 (86.58)	
**CV death:**			
Yes (%)	24 (0.46)	18 (1.89)	<0.001
No (%)	5160 (99.54)	936 (98.11)	

Abbreviations: BMI, body mass index; BP, blood pressure; CIMT, Carotid intima-media thickness; CV, cardiovascular; FBG, fasting blood glucose; HDL-C, high-density lipoprotein cholesterol; LDL-C, low-density lipoprotein cholesterol; MI, myocardial infarction; PAH, primary arterial hypertension, TC, total cholesterol.

**Table 2:** Multivariable Cox regression analysis for adverse outcomes.

Characteristic	CV Events		Stroke		MI		All-Cause Mortality	
	HR (95% CI)	*p*-Value	HR (95% CI)	*p*-Value	HR (95% CI)	*p*-Value	HR (95% CI)	*p*-Value
**Carotid plaque (yes)**	1.486 (0.865; 2.553)	0.151	1.02 (0.227; 4.585)	0.979	-	-	1.237 (0.174; 8.813)	0.812
**Hypertension only**	1.081 (0.719; 1.624)	0.709	0.987 (0.352; 2.736)	0.971	-	-	2.525 (0.618; 10.322)	0.197
**Hypertension with carotid plaque (yes)**	1.393 (0.927; 2.094)	0.111	1.172 (0.42; 3.271)	0.762	-	-	2.758 (0.674; 11.281)	0.158
**Carotid plagues on both sides (yes)**	1.203 (1.03; 1.406)	0.02	0.845 (0.527; 1.353)	0.482	2.222 (1.218; 4.0054)	0.009	1.588 (1.143; 2.206)	0.006

Abbreviations: CV, cardiovascular; HR, hazard ratio; MI, myocardial infarction.

## Data Availability

The original contributions presented in the study are included in the article, further inquiries can be directed to the corresponding author.
